# Predictors of Urinary Continence Recovery after Laparoscopic-Assisted Radical Prostatectomy: Is Surgical Urethral Length the Only Key Factor?

**DOI:** 10.3390/life13071550

**Published:** 2023-07-13

**Authors:** Alberto Ragusa, Aldo Brassetti, Francesco Prata, Andrea Iannuzzi, Pasquale Callè, Francesco Tedesco, Loris Cacciatore, Francesco Esperto, Giuseppe Simone, Roberto Mario Scarpa, Rocco Papalia

**Affiliations:** 1 Department of Urology, Fondazione Policlinico Universitario Campus Bio-Medico, 00128 Rome, Italy; alberto.ragusa@unicampus.it (A.R.); f.prata@unicampus.it (F.P.); andrea.iannuzzi@unicampus.it (A.I.); pasquale.calle@unicampus.it (P.C.); francesco.tedesco@unicampus.it (F.T.); f.esperto@policlinicocampus.it (F.E.); r.scarpa@policlinicocampus.it (R.M.S.); rocco.papalia@policlinicocampus.it (R.P.); 2 Department of Urology, IRCCS “Regina Elena” National Cancer Institute, 00144 Rome, Italy; giuseppe.simone@ifo.it

**Keywords:** urethra sparing, LARP, minimally invasive, urinary continence

## Abstract

Several efforts in recent years have been made to predict urinary continence (UC) recovery after radical prostatectomy. The aim of our study was to investigate the impact of surgical urethral length preservation (SULP) on urinary continence after LARP (laparoscopic-assisted radical prostatectomy). We retrospectively queried our datasets from May 2021 to May 2022. After the application of exclusion criteria, a total of 100 patients who underwent LARP for prostate cancer at our institution were enrolled. Through a sterile ruler inserted by a 12 mm trocar, the length of the membranous urethra spared during LARP was assessed intra-operatively. The baseline and peri- and postoperative data of patients were collected, and UC was defined as 0 or 1 on a safety pad. The median SULP was 20.5 mm (IQR, 14.5–25), and the median intraoperative EBL were 150 mL (IQR, 100–200). The Kaplan–Meier curve showed a significant difference at 20 mm, which was used as the cut-off value for SULP (log-rank test, *p* < 0.001). Multivariate Cox proportional hazards models showed that SULP and EBL < 250 mL were associated with UC recovery (all *p* < 0.02). Surgical urethral length preservation seemed to improve early UC recovery after LARP. Further multicentric studies are needed to confirm our findings.

## 1. Introduction

The standard of care for localized Prostate Cancer (PCa) is represented by Radical Prostatectomy (RP) in patients fit for surgery. Although in recent years, thanks to the introduction of minimally invasive surgical techniques, this approach has shown satisfactory oncological outcomes, Urinary Incontinence (UI) and Erectile Dysfunction (ED) still represent the sore notes of RP since they heavily weigh on men’s quality of life (QoL). Nowadays, Minimally Invasive Surgery (MIS) and, in particular, robotic surgery has been widely applied in order to improve the functional outcomes of RP. However, despite the several advantages of robotic and laparoscopic surgery, UI and ED still remain unsolved issues. Additionally, the continence pattern in the male patient was totally investigated on anatomical, functional, and surgical views to develop new surgical approaches to enhance Urinary Continence (UC) recovery. Regarding anatomical and functional aspects, the sphincteric system has a key role in antagonizing UI. Every attempt to preserve sphincters and particularly rabdosphincter must be performed to improve post-surgery UC; the inability to retain urine is a known factor that negatively impacts the QoL of both the patient and their partners.

Starting from the definition identified by the International Continence Society (ICS), UI is described as the involuntary loss of urine experienced during bladder storage [1]. 

The variety of definitions used for UI and the different methods for data collection have led to heterogeneous epidemiological data. 

Consequently, ICS’s reported values range between 1% and 49% for male UI. Among these patients, 40%–80% suffered from emergency UI, 10–30% were affected by a mixed male UI, and 10–15% were afflicted by male stress UI and, in this context, patients who underwent radical prostatectomy (RP) represented the prevalent part [2]. Since the prevalence of PCa among men and the high amount of RP due to an effective PCa-prevention, UI after prostate radical surgery described an actual problem impacting QoL.

Concerning surgical techniques, apical dissection represents a challenging step for oncological and functional outcomes. If, on one hand, reaching negative surgical margins delineated the crucial point of oncological surgery, on the other hand, preserving urethral tissue could impact the continence capacity and, consequently, the QoL of the patient.

Indeed, the length of the membranous urethra, due to its close relationship with the rhabdosphincter, represents the parameter that mainly influences Urinary Continence (UC) after RP in patients with an organo-confined tumor [3].

The length of the spared urethra is not the only factor affecting postoperative continence. Many other variables have been shown to have a significant impact on functional outcomes after radical prostatectomy: among these, the most studied were age, body mass index, prostate size, preoperative continence, the previous transurethral resection of the prostate, and a short membranous urethra at the baseline [4,5].

Although some studies showed an indirect correlation between the length of the urethra surgically spared during RP and UC after surgery [3], comprehensive and direct clinical data are still lacking.

The aim of this study was to explore the impact of the length of the membranous urethra when preserved during laparoscopic-assisted radical prostatectomy (LARP) and the UC rate.

## 2. Materials and Methods

### 2.1. Patient Population

Between May 2021 and May 2022, our dataset was queried for “localize PCa”, “LARP”, and “urinary continence”. Finally, baseline and peri- and post-operative data from 130 patients who underwent LARP for localized PCa at our institution were collected. All patients eligible for LARP were enrolled in the study. Exclusion criteria included locally advanced PCa (*n* = 12), previous UI (*n* = 5), previous prostate surgery (*n* = 3), or adjuvant radiotherapy for biochemical relapse (*n* = 10). All patients eligible for LARP were enrolled in this study. All patients included in the study provided written informed consent.

### 2.2. Procedures

**Figure 1:**
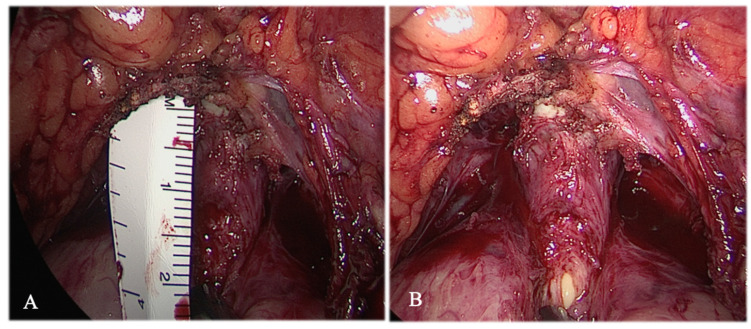
(**A**) Sterile ruler introduction and measurement from the prostatic apex to the entry of the urethra into the penile bulb; (**B**) Incision of the urethra.

All the LARPs were performed by the same experienced surgical team using a transperitoneal approach. Before the complete removal of the prostate, the prostatic apex was gently dissected using cold scissors, and the spared membranous urethra was measured as the distance from the prostatic apex to the entry of the urethra into the penile bulb through a sterile ruler, which was inserted by a 12 mm trocar intra-operatively, where the urethral caudal surgical cut was made (Figure 1). The vesicourethral anastomosis was now performed through a “single knot-single running suture” [6]; after passing both needles outside-in on the bladder neck at the 6 o’clock position without including bladder mucosa, the left stitch passed through the posterior musculofascial plate from right to left without including urethral mucosa. Then the suture passed outside-in into the full thickness bladder neck at the 7 o’clock position and subsequently inside-out in the urethra at the 7 o’clock position, including the mucosa layer. The left suture was passed again outside-in into the bladder neck at the 9 o’clock position. Likewise, the right stitch was passed in the same manner through the musculofascial plate from left to right and then into the bladder neck and urethra in a semicircular fashion, ranging from the 5 o’clock to 3 o’clock position. At this moment, gentle traction was performed on each thread to bring the bladder neck in contact with the urethra, allowing the insertion of an 18 F silastic catheter. The suturing of the anterior plate of the anastomosis began once the left stitch passed inside out at the 10 o’clock position and then outside-in at the 11 o’clock position in the urethra. Then, the left suture passed inside-out with the full thickness of the bladder neck. Thus, the right stitch was used to perform the same suture in a mirrored manner. The balloon was filled with 15 mL of saline water. Finally, the water tightness of the anastomosis was tested by irrigating 150 mL of the saline solution. 

### 2.3. Endpoints, Data and Statistical Analysis

The primary endpoint of this study was to evaluate UC recovery after LARP and evaluate its correlation with surgical urethral length preservation (SULP). The secondary endpoint was to identify predictors of UC recovery after LARP.

The urinary continence status was evaluated at 3, 6, 12, 18, and 24 months after surgery. UC was defined as 0 or 1 with a safety pad. Baseline patient characteristics and perioperative and pathological data included age, body mass index (BMI), a preoperative total prostate-specific antigen (tPSA), the day of the surgery, the number of months to achieve UC, estimated blood loss (EBL) during RP, the pathological T stage (pT), the surgical margin status and follow-up period. Regarding pre-operative evaluation, patients underwent a multi-parametric MRI examination of the prostate to identify a target zone for assessment during prostatic fusion biopsy whenever feasible. However, in cases where patients had pacemakers or other implanted metal devices, which contraindicated the use of multiparametric resonance, a saturation prostatic biopsy was conducted instead. 

BMI was calculated as the weight in kilograms divided by the height in meters squared (kg/m^2^). Urethral balloon catheters were removed on postoperative day 10 in all patients. All patients were instructed to perform pelvic floor training through Kegel’s exercises from 1 month after surgery until UC recovery, when achievable.

Continuous variables were presented as the median and interquartile range (IQR) and were compared using either the Wilcoxon rank sum test or Kruskal–Wallis one-way based on the normal or not normal distribution of the data, respectively (the normality of the distribution of the variables was assessed by the Kolmogorov–Smirnov test). Frequencies and proportions were used to report categorical variables, which were compared by means of Chi-squared. Continence recovery rate curves were plotted using the Kaplan–Meier method and were compared using the log-rank test. Predictors of UC were identified by means of univariable and multivariable Cox proportional hazards models in order to analyze the correlation between clinical parameters and continence recovery. The hazard ratio (HR) and 95% confidence interval (CI) were reported accordingly. The cut-off values for age, BMI, EBL, and spared urethral length were determined by exploratory research from the results obtained. All statistical analyses were performed using the STATA (StataCorp. 2021. Stata Statistical Software: Release 17. College Station, TX, USA: StataCorp LLC). A two-sided *p*-value < 0.05 was considered statistically significant.

## 3. Results

One hundred patients were included in our study. The baseline characteristics and peri-operative data are reported in Table 1.

**Figure 2:**
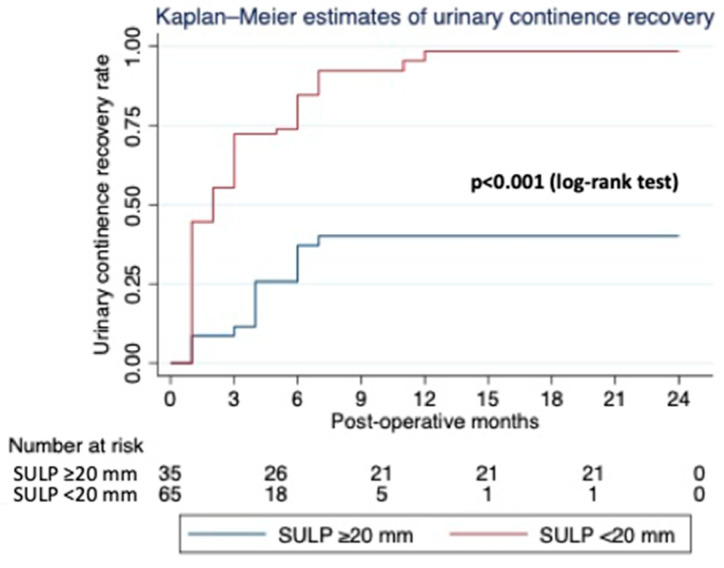
Urinary continence recovery rate curves plotted through the Kaplan–Meier method. A SULP ≥ 20 mm was associated with full continence recovery (*p* < 0.001, log-rank test).

The median age of patients, BMI, and preoperative tPSA were 64 years (IQR, 62–67), 27 kg/m^2^ (IQR, 24.6–29), and 6.4 ng/mL (IQR, 4.9–8.3), respectively. The majority of patients presented a pre-biopsy PI-RADS score of four (72%) and a pre-operative ISUP score of two (46%). Concerning peri-operative data, the median SULP was 20.5 mm (IQR, 14.5–25), the median intraoperative EBL was 150 mL (IQR, 100–200), while the majority of patients displayed pT2b at the final pathology (44%), and all of them had negative surgical margins. At a median postoperative follow-up of 16 months (IQR, 12–20), 78% of patients were continent irrespective of SULP. After exploratory research from the results obtained, we hypothesized that there was a difference in urinary continence when the urethra spared was around 20 mm. Thus, the Kaplan–Meier curve was drawn for the cumulative rates of UC and with respect to the urethra spared value in an exploratory manner; a significant difference was seen at 20 mm. Then, we decided to apply 20 mm as the cut-off value. A urethra spared of ≥20 mm resulted in statistical significance, influencing UC recovery (log-rank test, *p* < 0.001) (Figure 2). 

The cumulative continence recovery rates at 12 months were 97.9% for patients with SULP ≥ 20 mm and 43.5% for patients with SULP < 20 mm (Table 1). The univariate analysis of the Cox proportional hazards model showed SULP as a continuous variable (HR 1.08; 95% of CI (1.05–1.11; *p* < 0.001), BMI (HR 0.88; 95% CI 0.83–0.94; *p* ≤ 0.001), age (HR 0.92; 95% CI 0.89–0.96; *p* ≤ 0.001), and EBL (HR 0.99; 95% CI 0.98–0.99; *p* < 0.001) were significantly associated with UC recovery. The HR for SULP of ≥20 mm vs. <20 mm was 5.15 (95% CI 2.82–9.40; *p* ≤ 0.001), BMI < 30 vs. ≥30 kg/m^2^ was 8.57 (95% CI 3.10–23.70; *p* ≤ 0.001), age < 65 vs. ≥65 years was 5.87 (95% CI 3.15–10.92; *p* < 0.001), and for EBL < 250 mL was 5.73 (95% CI 2.29–14.31; *p* ≤ 0.001). The multivariate analysis of the Cox proportional hazards model showed that SULP (HR 1.05; 95% CI 1.01–1.08; *p* < 0.008) and EBL < 250 mL (HR 3.35; 95% CI 1.30–8.65; *p* < 0.012) were the only independent predictors of UC recovery (Table 2).

## 4. Discussion

Radical prostatectomy actually represents the standard of care for localized PCa in patients who are eligible for surgery. The technique of RP has been improved and evolved through the years from an open approach to more minimally invasive techniques with the aim of improving functional outcomes while preserving comparable oncological results. Nonetheless, common consequences of RP are represented by UI and ED, which could impact patients’ QoL. Between them, post-operative UI is one of the most bothersome complications after RP. If on the one hand, most of the patients who undergo RP experience UC recovery within one year; on the other hand, a good proportion of them suffer from stress UI indefinitely due to several factors. Actually, the processes of post-RP UI have not been well established, leading to the belief that multifactorial aspects have to be taken into consideration. At present, many studies have explored UI mechanisms after RP; however, the results appear to be controversial. Numerous surgical techniques have been suggested to maintain continence function. These techniques include nerve sparing, bladder neck preservation, urethral preservation, endopelvic-fascia preservation, the Retzius-sparing approach, and tension-free urethral anastomosis. Their aim is to safeguard and preserve continence during the surgical procedure.

Recent studies [7,8,9,10] have gradually unveiled the anatomical mechanism that is responsible for male urinary continence. In essence, this mechanism comprised two systems: the sphincteric system and the supportive system [11]. The sphincteric system encompassed the inner smooth muscle layer (comprising longitudinal and circular smooth muscle) as well as the striated urogenital sphincter muscle, also known as the rhabdosphincter. The rhabdosphincter stretches from the prostatic apex to the proximal bulbar urethra and is regarded as the crucial structure for maintaining urinary continence following radical prostatectomy. 

There is evidence that Endopelvic Fascia (EF) plays a role in urinary continence. According to a recent study conducted by Kwon et al., the preservation of this anatomical structure at the time of prostatectomy is an independent predictor of continence recovery (HR = 1.87; *p* = 0.002) [12]. Such evidence further supports the hypothesis that the best preservation/reconstruction of anatomical structures at the time of radical prostatectomy plays a key role in increasing the chances of achieving good functional outcomes.

The primary aim of RP is the complete eradication of cancer. It is crucial to ensure that no residual cancer remains after surgery, as this could necessitate further salvage therapies, which may introduce additional side effects for the patient and impose an additional financial burden on an already stretched healthcare system. For these reasons, the sparing of the urethra implicates a careful dissection and preservation of the structures that are responsible for continence without jeopardizing the patient’s oncological outcomes. Preserving a greater length of urethra potentially represents a key factor for UC recovery after surgery.

This relation between the urethra spared during RP and UC has become an area of active research and discussion within the urological community. Numerous studies have investigated the impact of different surgical techniques, including nerve-sparing approaches and variations in urethral preservation, on postoperative continence rates. Understanding the relationship between SULP and UC outcomes can lead to optimizing patient outcomes, enhancing their QoL following radical prostatectomy.

Urinary Incontinence represents one of the main long-term complications after RP, irrespective of the surgical approach considered, causing a deterioration in terms of patients’ QoL. Before surgery, individuals often experience elevated levels of fear and anxiety regarding the possibility of UI. As their symptoms improve, these distress levels typically decrease. However, if the restoration of continence is delayed, it can have a lasting detrimental effect on their QoL [13]. Studies have indicated that, on average, 16–51% of men continue to experience incontinence even 12 months after surgery, which can have a significant and enduring impact on both the patient and their partner [14].

Since membranous urethral length has represented the most significant predictor of UC, some authors proposed different paths to assess this factor by a Trans-Rectal Ultrasound (TRUS), pre-operative multiparametric-MRI (mp-MRI), or through direct intra-operative measurements.

In Y. Mizutani et al. series, membranous urethral length was assessed by TRUS during laparoscopic RP before and after the apex incision. The latter measurement seemed to impact the continence rate coded as 0 pads, assessing a urethral length of 2.6 ± 0.1 cm, 2.4 ± 0.3 cm, and 2.3 ± 0.2 cm after 1, 3, and 6 months follow-up, respectively [15]. Ultrasound imaging remained an operator-dependent technique, with all the limitations of this methodology.

Young Hwii Ko et al. showed that a pre-operative urethral length > 14 mm measured through mp-MRI was an independent predictor of UC at 30 days follow-up after robotic RP [3]. In a similar way using mp-MRI, F. Coakley et al. reported that a membranous urethra above 12 mm led to an achievement of UC after a 1-yr follow-up in 89% of patients. Instead, below 12 mm, this rate was found in 77% of cases [16]. Nevertheless, mpMRI measurements may be unreliable due to the pre-operative assessment of urethral length, without taking into consideration the potential impact of surgery on the urethra spared.

During surgery, there is a risk of damaging the proximal section of the membranous urethra through various maneuvers or cautery, which can lead to a decline in urinary function recovery. Philippe et al. conducted a study showing that the length of the membranous urethra after the operation played a role in early recovery from postoperative UI [17]. Additionally, Cho explained that the urethral length determined through a cystourethrogram after RP was more significant in relation to postoperative UI compared to the length calculated through preoperative MRI [18]. All these studies emphasized that relying solely on the preoperative measurement of membranous urethral length as a predictor for postoperative urinary incontinence in the era of minimally invasive RP could be precarious, as many other factors may contribute to UC recovery.

Regarding direct paths to assessing urethral length, A. Hakimi et al. located a ruler intraoperatively and evaluated it from the apex to the pubis symphysis during robot-assisted RP. In their series, a urethral length ≥ 20 mm was an optimal predictor for the ability to retain urine after 12 months from surgery [11]. Although this series is represented only in the current literature to assess the real intra-operative urethral length spared, it lacks comprehensive data.

In our study, patients were divided into two groups based on the SULP and according to Kaplan–Meyer in Figure 2. UC improved within a 1-yr follow-up in patients who had a SULP ≥ 2 cm in our series; then, no more improvements were recorded. However, the ability to retain urine was reached in almost all patients with such sparing (97.9%). A different course was registered in patients who had a SULP < 2 cm, showing an overall UC recovery of only 43.5%. Moreover, in the latter subgroup, the potential UC recovery was seen until 6 months after surgery; thereafter, no more patients improved their continence status.

SULP represented an important feature on the impact of incontinence after surgery; however, it was not the only factor in our series. Outcomes showed how SULP ≥ 2 cm, age < 65 years, BMI < 30 kg/m^2^, and EBL < 250 mL were all predictors of UC at univariate Cox regression analysis (all *p* < 0.001).

After multivariate Cox regression analysis, only SULP (*p* = 0.008) and EBL (*p* = 0.012) resulted in independent predictors of UC recovery. Although baseline data such as BMI and age may affect continence, as previously described in other studies [19,20,21,22,23], our results proved that a major impact on UC recovery might be represented by intra-operative factors. Considering that some known risk factors for post-operative UI, such as age or BMI, resulted in no longer producing significance 12 months after surgery could be potentially related to the damaged membranous urethra, with the latter predominant on baseline characteristics. 

The length of the urethra spared after PR surgery during apical dissection proved to be the parameter that most influenced UC in our series of patients with an organ-confined tumor [3] since rhabdosphincter preservation was better achieved through a conservative approach. In fact, in our population, there was an increase of 5% in the odds of UC after surgery for every mm of SULP (HR 1.05; 95% CI 1.01–1.08; *p* < 0.008). In addition, EBL could be an indirect predictor for the enhanced accuracy of prostatic apex dissection and, consequently, of gentle urethra preservation. The Santorini plexus, also known as the vesical venous plexus or the venous plexus of Santorini, is a network of veins surrounding the neck of the urinary bladder and the proximal part of the urethra. During apical dissection, the manipulation of the Santorini can potentially lead to bleeding from the plexus. Accuracy during this surgical step provides two achievements: the first is a minimization of blood loss, and the second is the maximization of the odds to preserve the urethra. Then, SULP is strictly connected to EBL, which is an indirect parameter of apical dissection accuracy.

As aforementioned before, the purpose of oncological surgery is to ensure the excision of the tumor while providing the best possible functional results. Urinary incontinence, as discussed earlier, is an extremely common condition after radical prostatectomy, which affects two-thirds of patients in the first 3 months after the procedure and persists in one out of ten males one year after surgery. There are multiple variables, both modifiable and non-modifiable, that are associated with the risk of urinary incontinence, and our study has confirmed that the length of the urethral stump is among these. Since the recent publication of the clinical study IMPROVE [XX–NCT02367404], it has been shown that there are no truly effective treatments to facilitate the postoperative recovery of continence [24]; the accurate selection of ideal candidates for radical prostatectomy becomes even more relevant. Therefore, comprehensively considering several factors, it is already possible to tailor the most suitable therapeutic path for the patient, taking into account his needs and expectations. In this perspective, the preoperative evaluation of urethral length (if it correlates with SULP) could represent a useful assessment when identifying the best candidates for prostatectomy.

Notwithstanding the interesting results obtained, this study was not devoid of limitations. First of all, the retrospective nature of the study, the relatively small sample size considered, and the expertise of surgical staff in LARP are all limitations. There is evidence supporting the fact that prostate volume plays a role in determining the chances of recovering urinary continence after prostatectomy. Due to the retrospective design of the present study, however, we were unable to retrieve information on the gland size of all patients included in the analysis, and this represents one of its main limitations. 

Second, due to the urethra-sparing technique, there was an increased risk of positive surgical margins that could jeopardize the oncological outcomes of our patient population. Effectively, the absence of positive surgical margins could be explained by the extensive experience of the first surgeon alongside all surgical staff for the LARP and urethra-sparing technique. Moreover, in order to obtain maximum sparing, the urethra was pulled and measured before sectioning. Whether SULP resulted from enhancement due to effective sparing or to a mechanical elongation remained unclear. 

## 5. Conclusions

These results suggest that an accurate apical dissection during LARP to improve the SULP can be associated with early UC recovery and, thus, with a better overall continence rate. Specifically, the maximal preservation of the urethra as well as gentle prostate apex control, considered as a lower EBL, were shown to be independent predictors of the UC status. Irrespective of patients’ characteristics, intraoperative surgical features and surgeon expertise seemed to represent the main drivers of continence status recovery in patients undergoing LARP.

## Figures and Tables

**Table 1:** Baseline demographic characteristics and peri-operative data.

Variable	Cohort (*n* = 100)
Age (years, median, IQR)	64 (62–67)
BMI (kg/m^2^, median, IQR)	27 (24.6–29)
tPSA (ng/mL, median, IQR)	6.4 (4.9–8.3)
Charlson Comorbidity Index (median, IQR)	5 (4–6)
Diabetes (n, %)	9 (9%)
Hypertension (n, %)	47 (47%)
PIRADS score (n, %)	
- 3	20 (20%)
- 4	72 (72%)
- 5	8 (8%)
ISUP (n, %)	
- 1	34 (34%)
- 2	46 (46%)
- 3	20 (20%)
- 4	-
- 5	-
pT stage (n, %)	
- pT2a	41 (41%)
- pT2b	44 (44%)
- pT2c	15 (15%)
- pT3	-
Estimated blood loss (mL, median, IQR)	150 (100–200)
Urethra length spared (mm, median, IQR)	20.5 (14.5–25)
Pads/Day at last control (n, %)	
- 0–1	78 (78%)
- 2–3	15 (15%)
- >3	7 (7%)
Continence rate	78%
Follow-up (months, median, IQR)	16 (12–20)

**Table 2:** Cox regression analysis to identify predictors of urinary continence recovery.

Variable	Univariable Analysis				Multivariable Analysis			
	HR	95.0% CI			HR	95.0% CI		
		Lower	Higher	*p* Value		Lower	Higher	*p* Value
Age (years)	0.92	0.89	0.96	<0.001	0.97	0.93	1.01	0.15
BMI (kg/m^2^)	0.88	0.83	0.94	<0.001	0.96	0.89	1.03	0.28
PI-RADS score	0.89	0.65	1.23	0.50	-	-	-	-
Diabetes	1.53	0.41	5.61	0.52	-	-	-	-
Hypertension	1.02	0.49	2.14	0.94	-	-	-	-
CCI	1.31	0.98	1.69	0.86	-	-	-	-
tPSA	1.02	0.96	1.08	0.50				
ISUP score	0.97	0.74	1.28	0.86	-	-	-	-
pT stage	0.90	0.72	1.12	0.36	-	-	-	-
EBL < 250 mL vs. >250 mL	5.73	2.29	14.31	<0.001	3.35	1.30	8.65	0.012
SULP (mm)	1.08	1.05	1.11	<0.001	1.05	1.01	1.08	0.008

## Data Availability

The data presented in this study are available on request from the corresponding author.
